# Comparison of outcome after stereotactic ablative radiotherapy of patients with metachronous lung versus primary lung cancer

**DOI:** 10.1186/s13014-023-02286-5

**Published:** 2023-06-07

**Authors:** Jonathan Benzaquen, Pierre-Yves Bondiau, Josiane Otto, Charles-Hugo Marquette, Jean-Philippe Berthet, Arash O. Naghavi, Renaud Schiappa, Jean-Michel Hannoun-Levi, Bernard Padovani, Jérôme Doyen

**Affiliations:** 1 Department of Pulmonary Medicine and Thoracic Oncology, Nice University Hospital, Pasteur Hospital, FHU OncoAge, Côte d’Azur University, 30, Voie Romaine, 06000 Nice, France; 2 CNRS, INSERM, Institute of Research On Cancer and Aging, Côte d’Azur University, Nice, France; 3 Department of Medical Oncology, Centre Antoine-Lacassagne, University of Côte d’Azur, Nice, France; 4 Department of Thoracic Surgery, Nice University Hospital, Pasteur Hospital, Nice, France; 5 Department of Radiation Oncology, H. Lee Moffitt Cancer Center and Research Institute, Tampa, FL USA; 6 Department of Radiology, Nice University Hospital, Côte d’Azur University, Nice, France

**Keywords:** Second lung cancer, Metachronous tumors, Stereotactic ablative radiotherapy

## Abstract

**Background:**

Early-stage lung cancer, primarily treated with surgery, often occur in poor surgical candidates (impaired respiratory function, prior thoracic surgery, severe comorbidities). Stereotactic ablative radiotherapy (SABR) is a non-invasive alternative that provides comparable local control. This technique is particularly relevant for surgically resectable metachronous lung cancer, in patients unable to undergo surgery.. The objective of this study is to evaluate the clinical outcome of patients treated with SABR for stage I metachronous lung cancer (MLC) versus stage I primary lung cancer (PLC).

**Patients and methods:**

137 patients treated with SABR for stage I non-small cell lung cancer were retrospectively reviewed, of which 28 (20.4%) were MLC and 109 (79.6%) were PLC. Cohorts were evaluated for differences in overall survival (OS), progression-free survival (PFS), metastasis-free survival, local control (LC), and toxicity.

**Results:**

After SABR, patients treated for MLC have comparable median age (76.6 vs 78.6, *p* = 0.2), 3-year LC (83.6% vs. 72.6%, *p* = 0.2), PFS (68.7% vs. 50.9%, *p* = 0.9), and OS (78.6% vs. 52.1%, *p* = 0.9) as PLC, along with similar rates of total (54.1% vs. 42.9%, *p* = 0.6) and grade 3 + toxicity (3.7% vs. 3.6%, *p* = 0.9). Previous treatment of MLC patients was either surgery (21/28, 75%) or SABR (7/28, 25%). The median follow-up was 53 months.

**Conclusion:**

SABR is a safe and effective approach for localized metachronous lung cancer.

## Background

Lung cancer is the leading cause of cancer death. Despite major progress in the understanding and management of this cancer, it is responsible for more than 33,000 deaths per year in France and 1.8 million deaths per year worldwide [1].

The standard treatment for localized non-small cell lung cancer (NSCLC) is parenchymal resection with lymph node dissection and there is increasing evidence in favor of minimally invasive surgical techniques to reduce operative risks and optimize functional recovery [2].

However, with higher average life expectancies and improved screening techniques (e.g. low dose thoracic computed tomography (CT)), there is an increased incidence of early stage I lung cancer diagnosed in the elderly or higher surgical risk patients [3]. Depending on the series, up to 25% of potentially resectable patients are not operable or refuse surgery [4, 5]. Notably, some patients will present with a second primary localized lung cancer after resection of their prior lung cancer, where operability can be challenging. These second cancers are defined as metachronous when they appear after a cancer free interval from the initial primary, as opposed to synchronous cancers, discovered concomitantly with the first. The cumulative incidence of metachronous lung cancer (MLC) is estimated at 8.3% at 5 years [6]. Although it is clearly accepted that MLC requires an ablative treatment (e.g. surgery, SABR, radiofrequency ablation), the choice of the therapeutic approach is debatable [7, 8] and role of non-surgical techniques needs to be further investigated.

Stereotactic radiotherapy and image-guided percutaneous radiofrequency ablation represent non-surgical ablative alternatives [9]. Stereotactic ablative radiotherapy (SABR) allows the delivery of high doses of irradiation according to a hypo-fractionated scheme. This non-invasive technique, with real time lesion monitoring and the possibility to follow respiratory movements, allows a 89 to 94% 2-year local control (LC) with a limited rate of severe toxicity [10, 11] for unresected stage I to IIA lung cancer. To our knowledge, eight studies have evaluated SABR treatment of a second lung cancer [12–19], of which only five included patients with metachronous tumors [12, 13, 16–18]. This is the first study to evaluate the safety and efficacy of SABR for stage I metachronous NSCLC compared to primary lung cancer, utilizing the date of current diagnosis to avoid survivor bias.

## Patients and methods

### Patient selection

This is a bicentric retrospective study including 137 patients treated with SABR between January 2007 and August 2016. The inclusion criteria were as follows: (i) patients with a stage IA or IB (TNM 8th edition [20]) NSCLC that is either pathologically confirmed or diagnosed at a multidisciplinary tumor board on the basis of positron emission tomography (PET) and thoracic computed-tomography (CT) if biopsy was not considered feasible; (ii) unresected stage I primary lung cancer (PLC) treated with SABR; (iii) patients previously treated by surgery or SABR for a primary stage I lung cancer and who are found to have a metachronous stage I lung cancer (MLC) treated by SABR; (iv) patients free of any metastasis or other malignancy other than cutaneous basal cell carcinoma; (v) patients with a curative oncologic plan; (vi) patients with at least 3 months of follow-up after SABR.

Patients with synchronous stage I primary tumors were excluded from this study.

The included patients were further divided into those who received SABR for a PLC (n = 109) and those who received SABR for a MLC (n = 28), MLC patients being excluded from the PLC group. MLC was defined as a primary cancer that appeared at least 4 years after the first lung cancer or at least 12 months after the first but in another lobe of the lung or had a different histology, according to the modified Martini criteria [21, 22].

In compliance with the declaration of Helsinki, the present study was approved by the French National Health Authorities (registration number: MR 004—n° F20210825142518). All families received written information on the study and gave their consent to the anonymous use of patients’ data for research purposes.

A signed free and informed consent was collected before administration of the SABR treatment.

### Treatment

Patients were immobilized during each SABR session using a custom-made vacuum mattress. Cyberknife® technology (Accuray, Sunnyvale, USA) was used for SABR. To account for tumor movement, patients either had an implanted gold fiducial into the tumor for tracking (91/137, 66.4%), an internal target volume (ITV) was created by a 4-dimensional CT scan (20/137, 14.6%), or direct soft tissue tracking in case of spontaneous tumor hyperdensity (26/137, 19%). Non coplanar beams using 6 MV photons were used and Ray tracing (Ray-trace effective path length) was utilized for dosimetric calculations. Dose constraints reported by the American Association of Physicists in Medicine (AAPM) Task Force 101 were used for SABR treatment [23]. The gross tumor volume (GTV) to clinical tumor volume (CTV) margin expansion was 0 mm, whereas the CTV to planning tumor volume (PTV) margin was expanded by 1–2 mm as low PTV margin are reported to be safe and to reduce toxicities in lung tracking condition [24]. The dose was prescribed relative to an isodose of 75–85% to cover the entire PTV volume with 95% of the prescribed dose, and corresponded to the International Commission on Radiation Units and Measurements (ICRU) Report 91 for stereotactic radiotherapy. The dose per fraction was reduced if the tumor was central (e.g. 5 potentially discontinued fractions of 10–12 Grays (Gy) instead of 3 consecutive fractions of 20 Gy), with protection of organs at risk was to be prioritized over target coverage.

### Clinical follow-up

Patients underwent thoracic CT and/or PET-CT within 2 months after the end of SABR treatment, and then every 3 to 6 months. Tumor response was assessed according to Response Evaluation Criteria In Solid Tumours (RECIST) 1.1 [25]. Time to event outcomes were defined from the last day of SABR to the event. Local (LC) and regional control (RC) were defined as a recurrence in the irradiated site and recurrence of ipsilateral thorax, respectively. Median follow-up was defined using reverse Kaplan–Meier method. Metastasis-free survival (MFS) was defined as any relapse outside the ipsilateral thoracic field. Progression-free survival (PFS) was determined by the time to any relapse (local, regional, or metastatic). Overall survival (OS) was defined as time to death from any cause. Toxicities were assessed according to the Common Terminology Criteria for Adverse Event (CTCAE) version 5.0. Patient clinical, toxicity, and survival data were obtained via chart review. Patients, their general practitioner, or oncologist were contacted to retrieve missing data.

### Statistical analysis

Qualitative data are represented as frequency, percentage, and 95% confidence interval. All continuous variables are reported by the median. Statistical comparisons were made using the Chi-square test for categorical data. Mann–Whitney test was used for quantitative variables. For the analysis of quantitative variables, thresholds were based on the median value of the variable. LC, RC, PFS, and OS were estimated and illustrated using the Kaplan Meier method and analyzed via log-rank. Patients were censored at death or last follow-up. Survival rates at different time points and 95% confidence intervals were also estimated. All statistical analyses were performed with a 5% alpha risk or 95% confidence interval using Statistical Package for the Social Sciences version 16.0.

## Results

### Patient characteristics

A total of 137 patients were irradiated with SABR, of whom 109 had stage I PLC and 28 had stage I MLC NSCLC. With a median follow up of 53 months, patients were primarily male (n = 99, 72.3%), Eastern Cooperative Oncology Group (ECOG) performance status 0 (n = 69, 50.4%), non-squamous histology (n = 53, 38.7%), and T1 stage (n = 83, 60.6%) (Table 1). The median age was 77.8 years with a 50 pack-year smoking history. The PLC and MLC group were relatively balanced without significant differences in patient characteristics. The majority of MLC patients had been previously treated with surgery for the first lung tumor (21/28, 75%). They were treated by SABR for the second lung tumor because of a medically inoperable condition (25/28, 89.2%). The remaining cases (3/28, 10.7%) were treated with SABR because of patient refusal of surgical management. Among MLC group, 12/28 (42.9%) patients presented at least 4 years of delay before the first lung tumor and the MLC, and 16/28 (57.1%) presented at least 1 year of delay of which 5/16 (31.2%) presented a different histology between the MLC and the first tumor, all of them developing the MLC in a different lung lobe than the first lung tumor. Of note, the histology was unknown for 17/28 (60.7%) MLC.

**Table 1:** Patient characteristics

	All	Primary lung cancer	Metachronous lung cancer	*p* value
Number of Patients	137	109 (79.6%)	28 (20.4%)	
Age (years)^a^	77.8 (57.1–96.6)	78.5 (57.6–96.6)	76.6 (57.1–88.9)	0.2
Follow-up (months)^a^	53 (38.6–67.4)	59.5 (40.2–78.7)	39 (31.3–46.7)	0.2
Gender				
Male	99 (72.3%)	81 (74.3%)	18 (64.3%)	0.3
Female	38 (27.7%)	28 (25.7%)	10 (35.7%)	
Smoking (pack-years)^a^	50 (0–180)	50 (0–180)	47.5 (0–140)	0.8
ECOG				
0	69 (50.4%)	52 (47.7%)	17 (60.7%)	0.2
1–2	68 (49.6%)	57 (52.3%)	11 (39.3%)	
Histology				
Squamous	38 (27.7%)	26 (23.9%)	12 (42.9%)	0.13
Non squamous	53 (38.7%)	45 (41.3%)	8 (28.6%)	
Adenocarcinoma		33 (30.3%)	4 (14.3%)	
Undifferentiated		11 (10.1%)	4 (14.3%)	
Unknown	46 (33.6%)	38 (34.9%)	8 (28.6%)	
TNM stage				
T1(N0M0)	83 (60.6%)	68 (62.4%)	15 (53.6%)	0.4
T1a			3 (10.7%)	
T1b			17 (60.7%)	
T1c			7 (25%)	
T2 (N0M0)	54 (39.4%)	41 (37.6%)	13 (46.4%)	
SABR indication				
Medically inoperable		103 (94.5%)	25 (89.2%)	0.3
Patient refusal		6 (5.5%)	3 (10.7%)	

^a^Median and range

### SABR treatment

The majority of patients were treated with 60 Gy in 3 fractions (n = 116, 84.7%), followed by five fractions regimens of 10 to 15 Gy (n = 21, 15.3%) (Table 2). The median radiation therapy duration was three days and only exceeded one week in 12 (8.8%) patients. The median interval between primary lung cancer to the second metachronous cancer was 39.4 months (13.9–121.4), which were initially treated with resection (n = 21, 75%) or SABR (n = 7, 25%) for their primary lung cancer. GTV and PTV values were significantly smaller (*p* = 0.007 and *p* = 0.001 respectively) for MLC when compared to the PLC cohort.

**Table 2:** Treatment characteristics

	All	Primary lung cancer	Metachronous lung cancer	*p* value
Total prescribed dose (Gy)^a^	60 (50–75)	60 (50–75)	60 (50–75)	0.8
Number of fractions^a^	3 (3–5)	3 (3–5)	3 (3–5)	0.7
Dose per fraction^a^	20 (12–20)	20 (12–20)	20 (12–20)	0.4
Radiation treatment duration (days)^a^	3 (3–14)	3 (3–14)	4 (3–8)	0.7
GTV (mL)^a^	8 (0.5–221.5)	9.3 (0.5–131.9)	5.1 (0.5–221.5)	0.007
PTV (mL)^a^	21.2 (2.8–167.3)	27.5 (2.8–167.3)	16.6 (3.8–35.9)	0.001
GTV (V100) coverage (%)^a^	100 (50–100)	100 (50–100)	100 (73–100)	0.6
PTV (V100) coverage (%)^a^	96 (40–100)	96 (40–100)	96 (65–100)	0.2
Number of beams^a^	130 (25–303)	131 (25–231)	116 (88–303)	0.4
Tracking technique				
Fiducial		72 (66.1%)	19 (67.9%)	0.3
Direct soft tissue tracking 4D		19 (17.4%)	7 (25%)	
CT scan ITV		18 (16.5%)	2 (7.1%)	
Primary diagnosis to second metachronous lung cancer (months)'			39.4 (13.9–121.4)	
First treatment of metachronous cancer				
SABR			7 (25%)	
Surgery			21 (75%)	

^a^Median and range

### Response and survival data

Overall, 8 (5.8%) patients had a complete response after SABR, 35 (25.5%) had a partial response, 55 (40.1%) had stable disease, 28 (20.4%) had progressive disease, and 11 (8%) patients could not be evaluated due to pneumonitis. Approximately half (n = 66, 48.2%) of patients recurred after SABR, with 30 (21.9%) having local recurrence, 56 (40.9%) having regional recurrence, and 54 (39.4%) having distant recurrence.

The 1-year/3-year LC and RC rate were 83.6%/72.6%, and 73.8%/58.2%, respectively. The 1-year/3-year MFS, PFS, and OS were 76.1%/58.9%, 68.7%/50.9%, and 78.6%/52.1%, respectively.

A total of 85 died (62%), with 7 (5.1%) from unknown cause, 51 (37.2%) experienced cancer specific mortality, 3 (2.2%) attributed to SABR toxicity, and 24 (17.5%) unrelated, of which consisted of 12 (50%) of respiratory failure, 8 (33.3%) of cardiac failure, 3 (12.5%) of cachexia, and 1 (4.2%) of fall trauma.

**Fig. 1:**
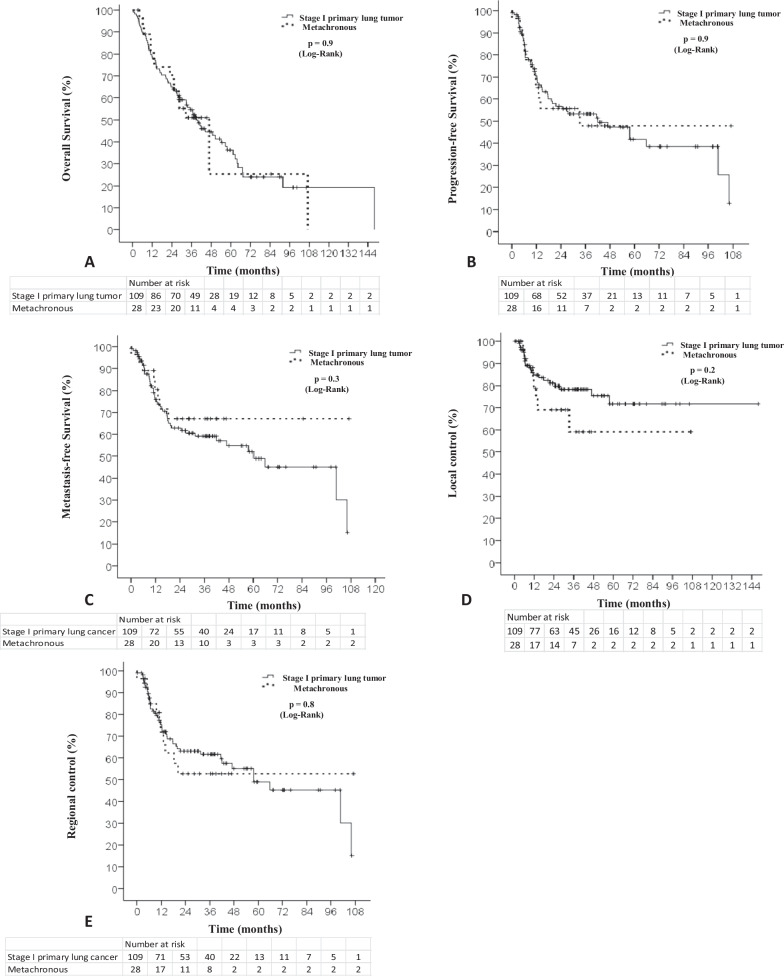
Survival analyses (Kaplan–Meier). **A** Overall survival, **B** recurrence-free survival, **C** metastasis-free survival, **D** local control, **E** regional control. Solid lines correspond to the stage I primary lung tumor group. Dashed lines correspond to the metachronous tumor group. Cross marks correspond to censored data

The median OS was 38 months in the PLC group versus 34 months for MLC (*p* = 0.9). There was a non-significant increase in cancer specific mortality in the PLC group (40.4% vs. 25%, *p* = 0.4), when compared to MLC. There was also no significant difference between PLC and MLC, in the 3-year PFS (68.7% vs. 50.9%, *p* = 0.9), MFS (76.1% vs. 58.9%, *p* = 0.3), LC (83.6% vs. 72.6%, *p* = 0.2) and RC (73.8% vs. 58.2%, *p* = 0.8) (Fig. 1).

Tumor volume is significantly different between the 2 cohorts and could consequently influence tumor control. With a cut-off of 8 mL (median), GTV was not found to be correlated with local control, locoregional control, metastasis-free-survival or progression-free survival (*p* > 0.05).

We divided the whole group in a group with small GTV (≤ 8 mL) and another one with larger GTV (> 8 mL). The prognostic impact of the cohort (primary versus metachronous) on local control was then analyzed in these 2 groups. No significant correlation was found (*p* = 0.5 if GTV ≤ 8 mL; *p* = 0.7 if GTV > 8 mL).

### Toxicities data

In the overall cohort, 71/137 (51.8%) patients experienced at least one toxicity attributed to SABR, with 66 (48.2%) grade 1–2 toxicity and 5 (3.6%) grade 3 + toxicity (Table 3).

**Table 3:** SABR toxicities

	Primary lung cancer (N = 109)			Metachronous lung cancer (N = 28)			*p* value		
	Total n (%)	Grade 1–2 n (%)	Grade > 2 n (%)	Total n (%)	Grade 1–2 n (%)	Grade > 2 n (%)	Total	Grade 1–2	Grade > 2
All Toxicities	59 (54.1%)	55 (50.5%)	4 (3.7%)	12 (42.9%)	11 (39.3%)	1 (3.6%)	0.59	0.55	0.99
Nausea	0	0	0	1 (3.6%)	1 (3.6%)	0			
Pneumonitis	4 (3.7%)	3 (2.8%)	1 (0.9%)	0	0	0			
Pneumothorax	15 (13.8%)	15 (13.8°%)	0	2 (7.1%)	2 (7.1%)	0			
Bronchopulmonary bleeding	1 (0.9%)	1 (0.9%)	0	0	0	0			
Chest wall pain	2 (1.8%)	2 (1.8%)	0	1 (3.6%)	1 (3.6%)	0			
Rib fracture	1 (0.9%)	1 (0.9%)	0	0	0	0			
Pleural effusion	6 (5.5%)	6 (5.5%)	0	1 (3.6%)	1 (3.6%)	0			
Lung fibrosis	12 (11%)	12 (11%)	0	3 (10.7%)	3 (10.7%)	0			
Atelectasis	6 (5.5%)	6 (5.5%)	0	1 (3.6%)	1 (3.6%)	0			
Dyspnea	12 (11%)	9 (8.3%)	3 (2.8%)	3 (10.7%)	2 (7.1%)	1 (3.6%)			

There were 3 (2.2%) grade 5 toxicities, 2 in the PLC cohort (1 radiation pneumonitis, 1 acute respiratory failure) and 1in the MLC cohort (acute respiratory distress).

There was no significant difference between the PLC and MLC groups in terms of total (54.1% vs. 42.9%, *p* = 0.6), grade 1 to 2 (50.5% vs. 39.3%, *p* = 0.6) and grade 3 + toxicities (3.7% vs. 3.6%, *p* = 0.9).

## Discussion

This study showed that SABR used in patients with unresected metachronous stage I lung cancer do not achieve different disease control and survival, when compared to stage I primary lung cancer, and do not lead to significant increase in toxicity. Matthiesen et al. [17] had also highlighted the value of this therapeutic approach in patients with a second lung cancer, but only one patient had a metachronous cancer in this cohort. There is also evidence that SABR treatment for metachronous cancer was associated with greater OS than patients treated for a synchronous cancer, but they did not compare survival data to a control population with stage I primary lung cancer [13].

Our data is consistent with prior prospective data, [18] which showed that patients treated with SABR for metachronous cancer had equivalent or even higher OS than those treated for stage I primary lung cancer. However, it should be noted that the OS of these patients were calculated from the primary cancer and not the diagnosis of their metachronous cancer, which causes a survivor bias. Since this bias is closely related to the stage of diagnosis, we chose to include only patients with stage I lung cancer. To our knowledge, only four studies evaluating SABR treatment of a second lung cancer analyzed survival from the second cancer rather than the first [14, 17–19], of which only 2 studies [17, 18] included patients with metachronous lung cancer. Studying this particular population is of major importance, indeed the treatment planning of previously irradiated patients represents a challenge regarding dosimetry constraints and patients’ therapeutic adhesion.

Of note, we underline an important limitation of this work related to the small number of patients in the MLC cohort which would require a larger cohort to strengthen these results and ensure the absence of statistical difference in outcomes of patients. Indeed, the local control curve in the MLC cohort appears to shift to the advantage of PLC cohort; an increase in study power could potentially reveal a statistical signal. In our study, local control of MLC was 72.6%, which is lower than a previous work [18] reporting at least 90.3% of local control in these patients, however the definition of metachronous patients included in this study is not as we defined in our study; indeed, we used a restrictive definition of metachronous patients.

The discovery of a lung nodule or mass in a patient previously treated for lung cancer is a situation that is not uncommon, with an incidence of a second lung cancer estimated to be between 1.5 and 3% per year per patient [26], especially in active smokers. If intra-lobar resection confers excellent results and is both therapeutic and diagnostic for operable patients [27], the discovery of a metachronous cancer in frail and elderly patients should suggest an indication of SABR with the aim of lung parenchyma conservation, as emphasized by the guidelines issued by ASTRO in 2017 [28].

Nevertheless, it is not always easy to distinguish between a metachronous primary cancer and a pulmonary oligometastasis, which commonly have worse prognosis [29] and may require the initiation of systemic therapy. Although the differential diagnosis between these 2 entities are complex [30, 31] and historically based on the criteria established in 1975 by Martini and Melamed [21], revised in 1995 [22] and then in 2007 by the ACCP recommendations [32], there is still no consensus as discussed by Fonseca and Detterbeck [33]. However, from a more pragmatic point of view, the treatment of a single pulmonary oligometastasis is in most cases an ablative treatment. Recently, it has been shown that molecular biological criteria obtained by sequencing would be superior to the criteria classically used to classify these entities [34]. Investigating circulating tumor DNA could represent an interesting perspective in order to assess new classifications’ biomarkers.

Differentiating MLC from lung oligometastases is challenging and may involve the risk of under-treatment of patients, yet the feasibility and efficacy of combining chemotherapy with SABR is demonstrated in patients developing a second lung cancer [35].

## Conclusion

In conclusion, our results show that SABR is safe and effective treatment for stage I metachronous lung cancer, with outcomes not significatively different to primary stage I lung cancer, with the proviso of a limited power in this present study. With over 4 years of follow-up, utilizing the follow-up from the diagnosis of the second metachronous cancer to avoid survivor bias, this study allows a fair representation between the long-term outcomes of primary versus metachronous NSCLC. However, these findings must be handled with the limitations related to the retrospective nature of our study, the bias of classification of oligometastatic versus metachronous lung cancer, and limited power due to a small number of patients with metachronous lung cancer.

Larger scale studies are required to confirm these results.

## Data Availability

Research data are stored in an institutional repository and will be shared upon request to the corresponding author.

## Abbreviations

SABR: Stereotactic ablative radiotherapy
MLC: Metachronous lung cancer
PLC: Primary lung cancer
OS: Overall survival
PFS: Progression-free survival
MFS: Metastasis-free survival
LC: Local control
NSCLC: Non-small cell lung cancer
CT: Computed tomography
AAPM: American Association of Physicists in Medicine
GTV: Gross tumor volume
CTV: Clinical tumor volume
PTV: Planning tumor volume
ICRU: International Commission on Radiation Units and Measurements
Gy: Grays
RECIST: Response Evaluation Criteria in Solid Tumors
ECOG: Eastern Cooperative Oncology Group
